# A retrospective analysis of the costs and management of genital warts in Italy

**DOI:** 10.1186/1471-2334-13-470

**Published:** 2013-10-09

**Authors:** Maria Michela Gianino, Sergio Delmonte, Emanuela Lovato, Morena Martinese, Sabrina Rondoletti, Maria Grazia Bernengo, Carla Maria Zotti

**Affiliations:** 1 Department of Public Health and Paediatrics Sciences, University of Turin, Via Santena 5 bis, Turin 10126, Italy; 2 STI Clinic of San Lazzaro Dermatological Hospital, A.O. Città della salute e della scienza, Via Cherasco 23, Torino 10126, Italy

**Keywords:** Condylomata acuminata, Human papillomavirus, Costs and cost analysis, Critical pathway

## Abstract

**Background:**

In Italy the prevalence of genital warts in women (15–64 years) is approximately 0.6% with an incidence of 0.4% per year. Treatments for GW are usually long, with moderate success and high costs. The aim of the study was to evaluate the diagnostic-therapeutic pathway, duration and setting of treatment, costs of episodes of condyloma in a population attending a regional STI clinic in Piedmont.

**Methods:**

This was a retrospective observational study conducted using medical records of outpatients who first visited the STI Clinic of San Lazzaro Dermatological Hospital in 2008. The patients’ medical histories were analysed for episodes that occurred and were cleared in 18 months following the initial visit. Data on screening methods for STIs, type of diagnosis for condyloma, treatment type, treatment setting, and anatomic lesion site were obtained from medical records. The costs were calculated for each episode.

**Results:**

A total of 450 episodes were analysed (297 men,153 women). The most frequently affected anatomic site was the genital area (74%) in both genders. With regard to treatment setting, 78.44% of patients received outpatient treatment at the STI clinic, 4% were treated at home, and 0.22% were hospitalised; 11.11% were treated in multiple settings. The mean number of treatments per episode was 2.03; although many patients received only 1 treatment (n = 207, 46%), exspecially cryotherapy or diathermy coagulation (64.73% versus 28.02% of episodes, respectively). The mean episode duration was 80.74 days. The mean cost (in 2011 euros) for an episode was €158.46 ± 257.77; the mean costswere €79.13 ± 57.40 for diagnosis and €79.33 ± 233.60 for treatment. The mean cost for treatment in a STI-Clinic setting was €111.39 ± 76.72, that for home treatment was €160.88 ± 95.69, and that for hospital care was €2825.94.

**Conclusions:**

The treatment of and associated costs for genital warts are significant. Several factors affect the cost, and internal STI clinic protocols, such as the 6 month window used to consider a recurrence or new diagnosis, create bias. Nonetheless, our findings how costs similar to those reported in the international literature and should be considered when deciding on which HPV vaccination programs should be provided by the public health system.

## Background

According to the Italian network of sexually transmitted infection (STI) sentinel clinic surveillance system, genital warts (GW), along with human papillomavirus (HPV) infections, are the most frequently occurring STI in Italy, representing 35.9% of STI diagnoses [1,2].

Genital warts are caused by HPV types 6 and 11, which are responsible for more than 90% of cases. In Italy, it has been estimated that the prevalence of genital warts in women between 15 and 64 years of age is approximately0.6%, with an incidence of approximately 0.4% per year [3]. The international literature estimates a similar range of 0.2%-0.7% in males and females [4-6]. In Europe, recent data show that every year, there are almost 600,000 new cases of genital warts, and these cases occur equally in both men and women.

Treatments for GW are usually long, with moderate success and high costs. The only effective prevention is vaccine protection against HPV types 6 and 11. Currently, two HPV vaccines are commercially available: Gardasil (Merck Frosst, Quebec, Canada) and Cervarix (GlaxoSmithKline, Brentford, UK). Both vaccines are highly effective in protecting against cervical cancer. However, only the quadrivalent vaccine has been shown to be effective against genital warts (GW) [7,8] in almost 100% of cases. The effectiveness of the quadrivalent vaccine has been demonstrated by real-life data in Australia, where GW incidence was reduced by nearly 94% among the vaccinated population in the 4 years after vaccination began [9].

In Italy, an anti-HPV vaccination campaign was introduced in 2007, mainly targeted against cervical cancer, among 12 year old girls. At the time, the high burden of disease (cervical cancer) in women in Italy and the high efficacy of the vaccine and safety data supported this decision. In addition, at that time, little was known by the scientific community in Italy concerning other HPV-related cancers and genital warts, and most official bodies recommended vaccination campaigns to primarly to target cervical carcinoma. Protecting against HPV types 16/18 (included in both vaccines) meant an estimated 70% reduction in cervical cancers in the years to come.

Genital warts are not a mandatory reportable disease in Italy. Therefore, there is a lack of registries, making it difficult to calculate the real impact of this disease. The only available data comes from the sentinel surveillance network of sexually transmitted infection clinics and from “ad hoc” studies. The Italian surveillance system for STI was established in 1991 [10,11] and involves collaboration with a network of public clinical centres that specialise in the diagnosis and treatment of STIs throught the country. Currently, there are 12 regional centres that collect data from a network of clinical centres that share diagnostic and therapeutic protocols. GW patients are difficult to follow; they do not tend to visit any specific specialist or clinic. STI clinics see only a small number of referred patients from other specialists or general practitioners. Most patients are treated outside of STI clinics, some as outpatients and some as inpatients, and treatment procedures vary greatly from medical to surgical treatments. Men tend to go to general practitionaires, urologists, andrologists or dermatologists. In contrast, women go mainly to gynaecologists or dermatologists. Patients usually require multiple visits - one study estimated an average of 5.7 visits for men and 6.3 visits for women [12] - and costs are usually high and highly variable. In Italy, costs range from 250 to 830 euros 5,6 [13].

Italy not only lacks definitive epidemiological data, which is mostly underestimated and biased by several factors, but it lacks estimates of the economic burden of disease. The aim of this study is to evaluate the diagnostic-therapeutic pathway and the costs of GW in one of the STI clinics situated in the region of Piedmont, Italy. This region has a population of 4 million, and more than 50% of the cases reported by the STI clinic regional surveillance network visit this particular clinic. STI clinic management in the Piedmont region follows these principles: service is accessible to all patients, services are offered free of charge, privacy of patients is guaranteed, and data collection is totally anonymous. This study aims to give an estimate of the cost of genital warts for the Italian health system.

## Methods

We carried out a retrospective analysis of outpatients who had their first visited the STI Clinic of San Lazzaro Dermatological Hospital, San Giovanni Battista Teaching Hospital, Turin in 2008. The patients’ medical histories for the 18 months following the initial visit were noted on their medical records.

An episode was defined by a panel of dermatologists as “any infection from the initial diagnosis to the clearance occurring during a period of 18 months”.

Clearance was defined as “the patient being free of disease or not coming back to the hospital for follow up-visits for 6 months after the last treatment”.

Therefore, an episode could include the occurrence of new lesions, the recurrence of previously treated lesions or new sites of infection within less than 6 months of last treatment.

The study focused only on the episodes; patients in concomitant treatment for other STIs were excluded, with the exception of HIV - positive patients.

The study protocol was approved by the Institutional Ethics Committee of San Giovanni Battista Teaching Hospital (Protocol 00009624 – 08 February 2010).

**Figure 1:**
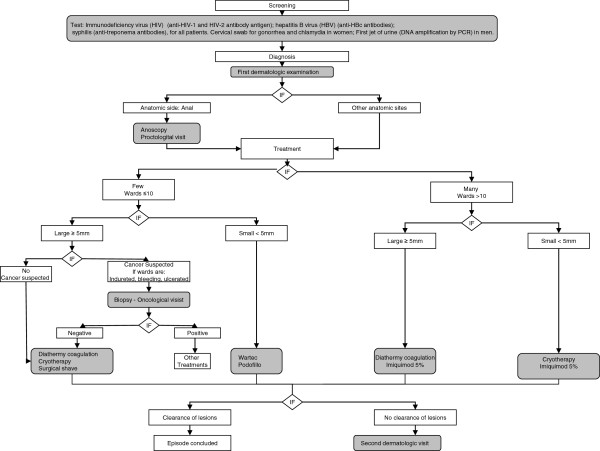
Flow diagram of clinical pathway of genital warts management.

According to the clinical pathway of GW management (Figure 1), we evaluated the methods of screening, the types of exams for diagnosis, and the types and numbers of treatments for each episode.

We also evaluated the anatomic sites of condyloma and the treatment setting. Anatomic sites of condyloma were classified as follows : genital (cervix, vagina, urethra, penis,or scrotum); anal (anus or perianal tissue); extragenital (groin, perineum or pubes); and multiple (any combination of at least two of the preceding). Treatment settings included the STI outpatient clinic, the home, the hospital or different combinations thereof.

The patients, on the basis of treatment numbers, were divided into seven classes: class 0 for patients who had undergone screening and diagnosis but not treatment; 1 for patients who had undergone screening and diagnosis and received treatment in a single visit; and 2, 3, 4, 5, and >5 for patients who had undergone screening and diagnosis and at least two treatments of the same or a different type.

The medical records were also searched for the mean duration of each episode of condyloma.

The cost analysis was performed from the perspective of the third-party payer (region of Piedmont) according to the breakdown shown in Table 1:

– the data on the hospitalization cost were taken from the 2011 hospitalization rates for Piedmont: DRG 267 with principal diagnosis code 07811 and intervention code 4904;

– the data on outpatient services were taken from the 2011 outpatient rates for Piedmont;

– the reference for the costs of pharmacotherapy was made to the pharmacy retail price.

**Table 1:** Cost breakdown

**Resource**	**Unit costs (€)**
Blood chemistry screening for:	
HIV (HIV-1 and HIV-2 antibody antigen)	€10.35
HBV (HBc antibodies)	€10.40
Syphilis (anti-treponema antibodies)	€7.80
Cervical swab for gonorrhea (detection by PCR assay)	€180.05
Cervical swab for chlamydia (detection by PCR assay)	€180.05
First jet urine (DNA amplification by PCR assay)	€180.05
Diagnosis*	
First dermatological examination	€30.00
Anoscopy	€32.50
Biopsy	€19.85
Proctological examination	€21.45
Oncological examination	€20.00
Second dermatological examination	€20.00
STI Clinic Treatments*	
Cryotherapy	€18.35
Diathermy coagulation	€18.35
Surgical shave	€40.65
Home Treatments (pharmacotherapy)**	
Imiquimod 5% (Aldara)	€ 76.56
Podofillotox (Wartec)	€28.40
Hospitalization treatments (DRG 267)***	€1371.72

* The data for costs were taken from the current rates for outpatient services (2011 - Piedmont Region).

** Pharmacy retail price.

*** Cost were taken from the 2011 hospitalization rates for Piedmont: DRG 267 with principal diagnosis code 07811 and intervention code 4904.

To calculate the mean costs we evaluated the cost of type of exams for screening and diagnosis and of type and number of treatment for each episode.

The costs for the various components, screening, diagnosis and treatment were stratified by patient sex and site of infection; the costs for diagnosis and treatment were also stratified by the setting of service delivery.

### Statistical analysis

All analyses were carried out using Stata 11 Version.

The quantitative variables were synthesized with the mean ± standard deviation (SD) and the median and interquartile range (IQR), while the continuous ones as mean and Standard Deviation (SD).

We examined: a) Anatomic site, duration and setting of treatment; b) Cost of condyloma episodes. To evaluate the possible associations between outcomes and categorical covariates, the Chi-square test were used. T-student test were used to compare continuous variables. Significance threshold was set at p < 0.05 (2-tailed) for all analyses.

## Results and discussion

The medical records of 502 outpatients (325 males, mean age 34.27 ± 11.62 years; 177 females, mean age 30.92 ± 10.98 years) were reviewed. At the end of the 18-month observation period, 52 patients were still under therapy and were therefore not included in the final analysis. There were 450 episodes of condyloma infection included in the study (89.64%).

### Anatomic site

**Figure 2:**
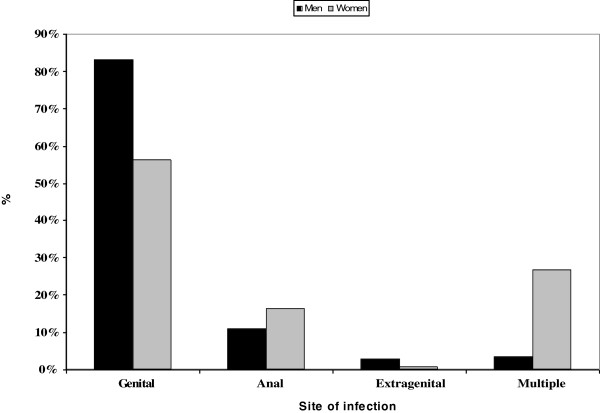
Percent of episodes of infection with condyloma in males and females stratified by site of infection.

The most frequently affected anatomic site was the genital area (74%), followed by the anus (12.67%) and multiple sites (11.33%). The least frequently affected site was the extragenital area (2%) (Figure 2).

The distribution of episodes by anatomic site differed between males and females (P < 0.001): the genital area was the most affected site (83.16% and 56.21% in males and females, respectively); the extragenital area was the least affected site (2.69% and 0.65% in males and females, respectively); the anus was more often affected than multiple sites in males (10.77% versus 3.37%), whereas the opposite was observed in females (16.34% versus 26.80%) (Figure 2).

### Treatments

With regard to treatment setting, 78.44% of patients received treatment at the STI clinic, 4% were treated at home, and 0.22% were hospitalised; 11.11% we treated in several different settings (9.33% at the STI clinic and at home; 1.11% at the STI clinic and in the hospital; 0.22% at home and in the hospital; and 0.44% at home, at the STI clinic and in the hospital); 28 (6.22%) received no treatment because of dropout. The mean number of treatments per episode was 2.03 (Table 2).

**Table 2:** Episodes classified by number of treatments and duration of episodes and stratified by sex, site of infection, and care setting

	**TOTAL**	**0 Treatment N. episodes (%)**	**1 Treatment N. episodes (%)**	**2 Treatment N. episodes (%)**	**3 Treatment N. episodes (%)**	**4 Treatment N. episodes (%)**	**5 Treatment N. episodes (%)**	**>5 Treatment N. episodes (%)**	**Mean no. of treatments (SD)**	**Mean duration of episode (days) (SD)**
**Total-N. = 450 (100%)**	**450***(100)*	**28 (6.22)**	**207 (46.0)**	**99 (22)**	**56 (12.44)**	**31 (6.89)**	**14 (3.11)**	**15 (3.33)**	**2.03 (1.34)**	**80.74 (92.13)**
**Gender**										
Men	297 *(100)*	18 (6.06)	130 (43.77)	70 (23.57)	41 (13.80)	21 (7.07)	8 (2.69)	9 (3.03)	2.05 (1.30)	79.79 (94.74)
Women	153 *(100)*	10 (6.54)	77 (50.33)	29 (18.95)	15 (9.80)	10 (6.54)	6 (3.92)	6 (3.92)	2.00 (1.41)	82.60 (87.14)
**Site**										
Genital	333 *(100)*	17 (5.11)	161 (48.35)	73 (21.92)	41 (12.31)	23 (6.91)	7 (2.10)	11 (3.30)	2.22 (1.68)	72.78 (88.30)
Anal	57 *(100)*	8 (14.04)	19 (33.33)	15 (26.32)	7 (12.27)	3 (5.26)	4 (7.02)	1 (1.75)	2.87 (2.09)	125.14 (108.38)
Extragenital	9 *(100)*	1 (11.11)	5 (55.56)	1 (11.11)	1 (11.11)	1 (11.11)	0 (0)	0 (0)	2.23 (2.06)	66.33 (95.40)
Multiple	51 *(100)*	2 (3.92)	22 (43.14)	10 (19.61)	7 (13.73)	4 (7.84)	3 (5.88)	3 (5.88)	2.47 (1.78)	85.55 (84.30)
Setting*										
STI Clinic	353 *(100)*		194 (54.96)	81 (22.95)	39 (11.05)	22 (6.23)	9 (2.55)	8 (2.27)	1.85 (1.22)	75.20 (86.0)
Home	18 *(100)*		13 (72.22)	4 (22.22)	0 (0)	1 (5.56)	0 (0)	0 (0)	1.39 (0.78)	31.11 (47.26)
Hospital	1 *(100)*		0 (0)	1 (100)	0 (0)	0 (0)	0 (0)	0 (0)	2.00 (0)	9.00 (0)
STI Clinic and Home	42 *(100)*		0 (0)	11 (26.19)	14 (33.33)	6 (14.29)	4 (9.52)	7 (16.67)	3.57 (1.42)	156.26 (117.57)
STI Clinic and Hospital	5 *(100)*		0 (0)	2 (40.0)	2 (40.0)	1 (20.0)	0 (0)	0 (0)	280 (0.84)	150.00 (80.62)
Hospital and Home	1 *(100)*		0 (0)	0 (0)	0 (0)	0 (0)	1 (100)	0 (0)	5.00 (0)	288.00 (21.21)
STI Clinic and Hospital and Home	2 *(100)*		0 (0)	0 (0)	1 (50.0)	1 (50.0)	0 (0)	0 (0)	3.50 (0.71)	259.00 (21.21)

*SD*, Standard Deviation; *IQR*, Interquartile range.

* only patient with treatment.

Forty-six percent of patients received 1 treatment (n = 207); the number of patients decreased as the number of treatments increased up to 5 treatments, after which the number of treatments increased in parallel with the number of patients who had received more than 5 treatments (3.33%) (Table 2). This trend was similar for both sexes, and according to anatomic site, except for the anal area (Table 2). There was no statistically significant difference in the mean number of treatments between males and females (P = 0.43) or in the anatomic site of condyloma (P = 0.23).

The most frequent type of therapy was 1 treatment with cryotherapy or diathermy coagulation (64.73% versus 28.02% of episodes); pharmacotherapy was much less frequent (approxximately 6.28% of patients).

In patients who received 2 treatments, the majority (54.55%) underwent 2 sessions of cryotherapy, followed by a combination of diathermy coagulation and cryotherapy (14.14%). Among patients who received between 3 and 4 treatments, most received multiple treatments of cryotherapy alone. No predominant sequence of treatment was observed in patients who received more than 4 treatments.

Analysis of the number of treatments per setting showed that the mean number was higher when patients were treated in a combination of different settings, except for the hospital and home combination, as this combination involved only 1 patient. The lowest number of treatments was observed in patients treated at home (Table 2). There was a statistically significant association between the number of treatments and treatment setting (P < 0.001).

The mean episode duration was 80.74 days. No statistically significant difference in mean duration was observed between males and females (P = 0.363) or among anatomic sites (P = 0.048); whereas, there was a statistically significant difference in mean duration according to the setting (P < 0.001): the mean duration was longer for patients treated with a combination of different treatment settings (Table 2).

### Cost of condyloma episodes

The mean cost (diagnosis and treatment) for an episode of condyloma was €158.46 ± 257.77: the mean cost of diagnosis was €79.13 ± 57.39, and that of treatment was €79.33 ± 233.60.

**Figure 3:**
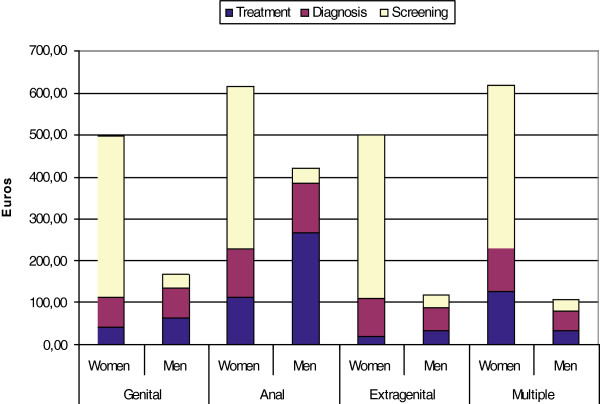
Mean cost (in 2011 Euros) of diagnosis ,treatment and screening per condyloma episode stratified by gender and infection site.

The mean costs of screening, diagnosis and treatment of a condyloma episode, stratified according to sex and site of infection, are shown in Figure 3.

There was a statistically significant difference in the mean cost between females and males (€161.35 ± 267.30 versus €156.97 ± 253.17) (P = 0.008) and in the mean cost according to site of infection (P = 0.0001): anal condylomata were the most expensive, and extragenital sites of infection the least expensive.

When the cost of screening (€152.58 ± 170.46) was added to the cost of diagnosis and treatment, the mean cost per episode was €311.04 ± 310.54: the mean cost of screening was €386.30 ± 29.11 for females and €32.19 ± 29.37 for males.

The mean cost (diagnosis and treatment) in the STI-Clinic setting was €111.39 ± 76.72; the costs for home (€160.88 ± 95.69) and hospital care (€2825.94) were higher. The mean cost for patients treated in a combination of different settings was progressively higher starting with STI clinic and home (€273.37 ± 146.40), then STI clinic and hospital (€1565.25 ± 52.43), and finally hospital and home (€1886.36) (Table 3).

**Table 3:** Mean cost (in 2011 Euros) of diagnosis and treatment of a condyloma episode stratified by care setting

**SETTING**	**Diagnosis**^**b **^**(SD)**	**Treatment**^**b **^**(SD)**	**Total Diagnosis + Treatment**^**b **^**(SD)**
STI Clinic	75,06 (54,21)	36,33 (29,61)	111,39 (76,72)
Home	57,22 (36,29)	103,66 (61,98)	160,88 (95,69)
Hospital	82,50 (−)	2.743,44 (−)	2825,94 (−)
STI Clinic and Home	119,07 (67,84)	154,31 (86,60)	273,37 (146,40)
STI Clinic and Hospital	160,50 (53,19)	1.404,75 (15,35)	1.565,25 (52,43)
Hospital and Home	135,00 (−)	1751,36 (−)	1.886,36 (−)
STI Clinic and Hospital and Home	241,25 (93,69)	1.504,91 (54,14)	1.746,16 (147,83)

^b^ Only patients who received treatment (N.422).

*SD*, Standard Deviation.

## Conclusions

The aim of this study was to examine the diagnostic-therapeutic pathways of a cohort of patients with condyloma and to determine the cost for a single episode. This approach distinguishes our study from others that investigated either the total cost of infection with condyloma over one or more years or the mean annual cost of condyloma diagnosis and treatment in cases that had or had not yet cleared [12,14-18]. During the 18-month observation period, we noted 450 episodes (89.64% of medical records reviewed). The mean duration of an episode was 80.74 days; this finding is in line with previous observations by Hoy (95.4 days) and Marra (69.3 days) [19,20].

Similar to a study by Insinga [21] but different from other studies [19,20] our findings showed that the mean duration of an episode of infection with condyloma was longer for females than for males. This difference might have been observed because might have been observed because women in Italy usually seek care from their private gynaecologists and visit an STI clinic only in more complicated cases.

The mean number of treatments was similar to that reported elsewhere: 2.4 by Marra [20] and 2.2 by Hoy [19]. The mean number of treatments was slightly higher for males than for females; our range (2,05 for males versus 2,00 for females) was narrower than that found by Hoy (2.5 versus 1.9) [19].

There was a marked predominance of monotherapy, with cryotherapy being the treatment most often utilised. Differing from the findings reported by Van Der Meijden [14] our study revealed a low prescription rate for podophyline (Wartec).

The majority of episodes were treated in a single setting, the STI clinic, where most infections with condyloma were cleared with a single treatment.

Treatment with hospitalisation occurred in 1.2% of episodes, significantly fewer than that reported by Merito (12.6%) [15] because most of the episodes in our study were treated by cryotherapy delivered in the STI clinic. Home treatment was also far less frequent in our study than in that by Merito (14% versus 36.5%) [15] as there was a low level of podophyline prescription in our study.

The mean cost of diagnosis and treatment per episode was €158.46, which is fairly similar to that reported by Marra (€133.68 at 2006 exchange rates) 20 and Desai (€130.20 at 2011 exchange rates) [13] but far less than those reported by Hoy (€520.14 at 2004 exchange rates) [19] or Insinga (€472.07 at 2000 exchange rates) [21] The likely reason for this difference is that the latter two studies refer to private health care plans that differ substantially from the Italian national health system in the delivery of services, costs of services and type of treatment given. Insinga [21] for example, reported that approximately 40% of patients received drug therapy, compared to 9.6% of those in our cohort, and that the mean cost of a visit was €118, compared to €30.00 in our study. Costs of care of GWs were reported from the United Kingdom (€ 363.93 for males and € 346.31 for females) [12] and the Netherlands (€ 221.34 for males and € 292.29 for females) 14 in 2000, from Spain (€ 832.50) [6] and France (€ 342.40) [5] in 2005 and from Ireland (€ 335) [18] in 2007. Comparisons with our cost of management per episode are not straightforward because of differences in target and setting studied and in episode definitions.

The mean cost per episode of infection with condyloma was higher for females than for males, which is in line with previous studies of health care systems similar to that of Italy [20,21] but contrasts with studies conducted in the United States, where the mean cost per episode was found to be higher for males than for females. One of the factors that drives up the cost per episode in females is the cost of diagnosis, most likely because more females than males present with anal condyloma (16.34% versus 10.77%) and multiple sites of infection (26.80% versus 3.37%), which require more costly procedures such as anoscopy. Furthermore, anal warts incur higher costs because they require more treatments and take longer to clear. Episodes of infection treated at an STI clinic are the least expensive, whereas those treated in the hospital are the most expensive. This differenceis confirmed by a previous study in Italy in which a higher number of hospitalisations increased the mean annual cost per patient [15].

Our study has several limitations. The first limitation is that the study was conducted at only one of the region’s STI centres, although this centre receveid more than 50% of the condyloma infections. The second limitation is that our study enrolled only cases diagnosed by the public health system, and could be missing patients who sought diagnosis and treatment from private providers. As shown by the data reported by Insinga [21] the number of patients who visited private providers may shift the economic burden of infection with condyloma.

The third limitation is that our study focused only on episodes that had cleared during the 18-month observation period. Having a longer observation period would permit the enrolment of patients with episodes of longer duration, which would consequently increase the mean cost of care.

In conclusion, our study shows that the treatment of genital warts and the associated costs are significant. Among other factors, this burden and cost should be considered when deciding on which HPV vaccination programs should be provided by public health systems. Due to the limitations listed above, our results are likely to have underestimated the number of cases and economic burden of condyloma.

### Highlights

We performed a retrospective analysis of medical records of outpatients with condyloma and obtained the following results:

•For each episode, the mean duration was 80.74 days and the mean number of treatments was 2.34.

•The most frequent treatment setting was STI clinic.

•The mean cost for an episode of condyloma was €158.46.

•The mean costs of diagnosis and treatment were €79.13 and €84.59, respectively.

## Abbreviations

STI: Sexually transmitted infection; GW: Genital warts; HPV: Human papillomavirus; HIV: Human immunodeficiency virus; SD: Standard deviation; IQR: Medians and interquartile range.

## Competing interests

The authors declare that they have no competing interests.

## Authors’ contributions

MMG, SD, SR, MGB and CMZ designed the study; MM, EL recorded patient data in a database; MMG, EL and MM conducted the analysis. MMG has full access to all of the data in the study and takes responsibility for the integrity of the data and the accuracy of the data analysis. All authors read and approved the final manuscript.

## Authors’ information

CMZ Associate Professor, Department of Public Health – University of Torino.

MGB Full Professor, University of Torino - STI Clinic of San Lazzaro Dermatological Hospital, A.O. Città della salute e della scienza.

MMG Associate Professor, Department of Public Health – University of Torino.

SD Medical Doctor, STI Clinic of San Lazzaro Dermatological Hospital, A.O. Città della salute e della scienza.

SR Medical Doctor, STI Clinic of San Lazzaro Dermatological Hospital, A.O. Città della salute e della scienza.

MM Medical Doctor in Training, Department of Public Health – University of Torino.

EL Medical Doctor in Training, Department of Public Health – University of Torino.

## Pre-publication history

The pre-publication history for this paper can be accessed here:

http://www.biomedcentral.com/1471-2334/13/470/prepub
